# Forensic Point-of-Care Test to Unveil the Cause of Death in Sudden Cardiac Deaths: A Case Series

**DOI:** 10.7759/cureus.84513

**Published:** 2025-05-20

**Authors:** Sravan JS, Sibi Vijayakumar, Suhail Alikkal, Arneet Arora

**Affiliations:** 1 Forensic Medicine and Toxicology, Pacific Medical College and Hospital, Udaipur, IND; 2 Forensic Medicine and Toxicology, Sri Ramachandra Institute of Higher Education and Research, Chennai, IND; 3 Forensic Medicine and Toxicology, All India Institute of Medical Sciences, Bhopal, Bhopal, IND

**Keywords:** cardiac biomarkers, ck-mb, forensic biochemistry, forensic medicine, forensic pathology, legal medicine, point-of-care testing (poct), sudden cardiac death, troponin i

## Abstract

This forensic case series from the central region of India, to the best of our knowledge, signifies a paradigm shift, leveraging point-of-care testing (POCT) to swiftly unravel the mysteries of sudden cardiac deaths. The autopsy series integrates the SD Biosensor F2400 Analyzer (Suwon, South Korea) device and POCT cartridges, centering on cardiac troponin I (cTnI) and creatine kinase-myocardial band (CK-MB). Across four diverse cases, intricate details emerge, weaving a complex tapestry of trauma and cardiovascular pathology.

In Case 1, a fatal road accident unveils air embolism and head injury intricacies. Case 2 delves into breathlessness, exposing myocardial infarction complexities. Revisiting Case 3 reveals sudden collapses' challenges, emphasizing acute cardiac nature through cTnI and CK-MB values. Case 4 intertwines head injury complexities with cardiac involvement, accentuating the delicate balance between trauma and cardiac pathology.

The discussion navigates through case nuances, underlining the cost-effectiveness and time efficiency of POCT. Comparative analyses with existing literature showcase POCT's uniqueness in autopsy examinations. Challenges in discerning antemortem changes from postmortem alterations underscore the need for meticulous analysis. In conclusion, this series showcases the potential of POCT in revolutionizing forensic pathology and providing rapid, reliable diagnostic insights into sudden cardiac deaths.

## Introduction

Forensic pathology presents unique challenges in diagnosing cardiovascular causes of sudden death, with myocardial infarction being one of the most significant. Recent advances in diagnostic medicine need to be incorporated, and innovations are important to rapidly and accurately identify the cause and manner of death [1]. Conventional diagnostic tests cannot always be reliable, especially during the first few hours following ischemia. Also, the macroscopic and routine histological changes may be absent or minimal during this time period. Cardiac biomarkers such as cardiac troponin I (cTnI), cardiac troponin T (cTnT), and creatine kinase-myocardial band (CK-MB) are well-established in clinical medicine for detecting ischemic heart diseases. However, they have not been effectively implemented in autopsy practice [2]. For example, in clinical settings, cTnI values are typically considered elevated when above 0.03 ng/mL, indicating myocardial injury [3].

Point-of-care testing (POCT) provides rapid, near-site diagnostic results without relying on centralized laboratory infrastructure. In forensic settings, it has emerging utility in supporting cause of death determination, especially when histopathology is unavailable or inconclusive. As such, the current autopsy series, conducted in a tertiary care facility in India, presents a novel approach to using POCT for cardiac biomarkers in sudden death investigations. This case series will be useful to support forensic pathologists by demonstrating how POCT can assist in diagnosing acute myocardial infarction (AMI) where traditional evidence is not available. This case series has four examples in which POCT contributed to the interpretation of cardiovascular pathology and also highlighted its potential as a supplementary diagnostic tool in forensic pathology.

## Case presentation

Case 1

A 37-year-old woman who sustained serious head trauma following a road traffic accident died roughly 36 hours after the incident. The autopsy examination which was carried out around 16 hours after death, revealed antemortem head injury with evidence of air embolism in dural venous sinuses as the cause of death. Some of the relevant autopsy findings consisted of a comminuted skull fracture with underneath epidural hematoma, subdural hematoma, and contusion necrosis of the brain on the frontal and temporal lobe on the left hemisphere. Air embolism was detected in dural venous sinuses of meninges and also in the heart chambers with a rough estimate of about 100 ml of air in the circulatory system. Petechial hemorrhages were seen on the posterior aspect of the heart, the base of the aorta, and both auricles. The coronary arteries were patent. On POCT examination on peripheral venous blood, cTnI POCT was found to be 17 ng/mL, and CK-MB POCT was found to be 123.2 ng/mL.

Case 2

A 66-year-old man was admitted to a tertiary care center, who presented with shortness of breath and loose stool. Within a couple of hours after admission, the patient collapsed and succumbed to death even after adequate resuscitation efforts. Postmortem examination was done 10 hours after death. The cTnI POCT and CK-MB POCT were measured on a venous blood sample obtained at the commencement of autopsy from the right femoral vein and were markedly elevated (19.5 ng/mL and 97.5 ng/mL respectively). Anatomical dissection of the heart during autopsy revealed complete occlusion of the left anterior descending (LAD) artery. The right coronary artery (RCA) also showed 25-50% occlusion of the lumen for a length of 2 cm starting at the origin of the artery. The left circumflex artery (LCx), had athermanous changes but did not show any occlusion of the lumen throughout its course. Myocardium had dark red staining which suggests a recent infarct in the interventricular septum (IVS) and the anterior wall of the left ventricle which also addressed the very high values obtained in POCT. Thus the cause of death was attributed as myocardial infarction as a result of coronary artery occlusion.

Case 3

**Figure 1:**
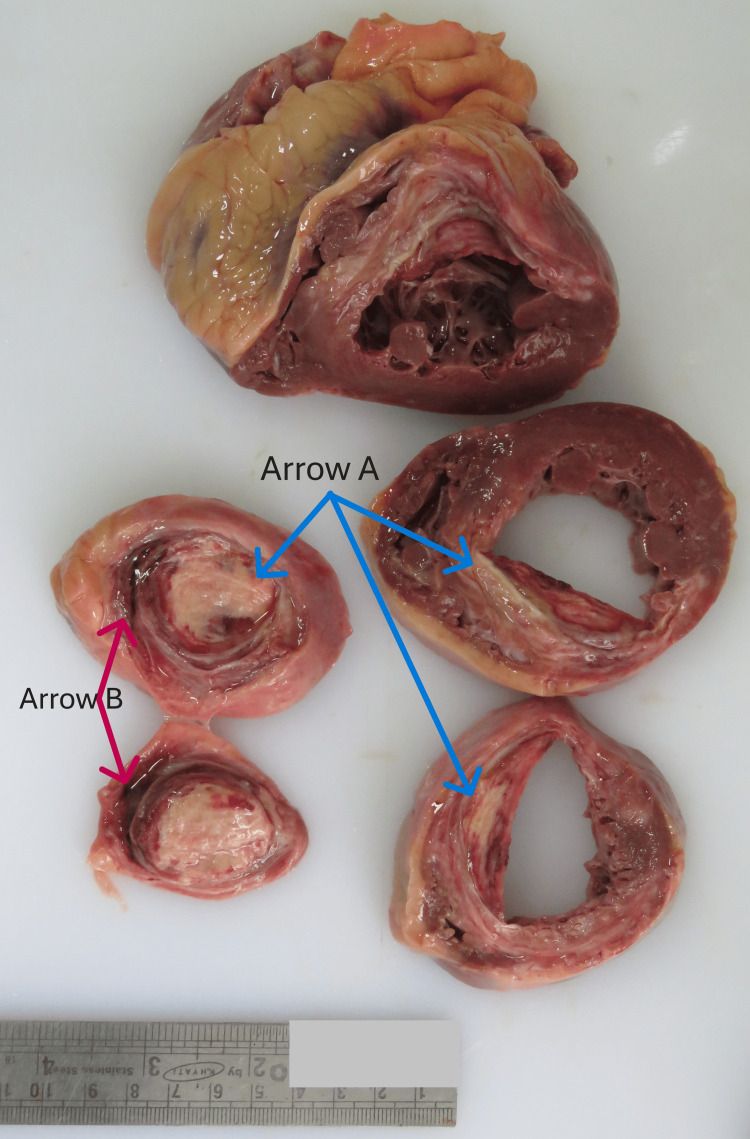
Figure showing gross findings in cut sections of heart suggestive of old and recent myocardial infarction as seen during the autopsy examination of Case 3. Arrow A shows the whitish myocardium suggestive of old myocardial infarction. Arrow B shows the reddish myocardium suggestive of recent myocardial infarction.

A 53-year-old man was found unconscious at his home and was shifted to the hospital. At the hospital, the person was declared brought dead and a postmortem examination was ordered to rule out any foul play as the death was unattended. Postmortem examination was done 14 hours after death and revealed an extensive picture of cardiovascular pathology. There was a marked occlusion of the left anterior descending artery along with myocardial bridging. A whitish fibrous myocardium was present over the apex of the left ventricle and interventricular septum near the apex, suggestive of old myocardial infarction (refer to Figure 1, arrow A). Along with that, a reddish discoloration of the interventricular septum starting at 2 cm above the apex (refer to Figure 1, arrow B) was visualized, suggestive of a recent myocardial infarction. The autopsy surgeons had to get confirmation regarding the cause of death since the victim collapsed suddenly and with no warnings or any history of previous cardiac illness even though the anatomical heart dissection revealed both old and recent myocardial infarction. Here also the POCT examination of the right femoral venous blood was crucial in alleviating any doubt regarding the cause of death and gave the necessary cross-validation. The POCT of case three revealed values of cTnI to be greater than 20 ng/mL and CK-MB value to be greater than 200 ng/mL. These POCT values denote that the actual value was much more than the quantifiable limit of the machine.

Case 4

A 65-year-old man succumbed to a head injury 15 days after a fall from stairs. Postmortem examination carried out 14 hours after death showed a comminuted skull fracture, extradural hematoma, subdural hematoma, and a diffuse subarachnoid hemorrhage. In addition, contusion necrosis of the cerebral lobes was seen and there were multiple rib fractures. Cardiac examination showed 80% LAD occlusion over a distance of 6 cm and 20% LCx occlusion over a distance of 2 cm starting from the origin of these arteries. Here the cTnI POCT was recorded at 0.25 ng/mL and CK-MB POCT was 155.9 ng/mL from the peripheral venous blood of the deceased.

Thus, all four cases have different histories and clinical pictures and also show different scenarios of cTnI and CK-MB values (Table 1).

**Table 1:** Case details with cardiac troponin I (cTnI) and creatine kinase-myocardial band (CK-MB) point-of-care test values.

	Age	Sex	cTnI (ng/mL)	CK-MB (ng/mL)
Case 1	37 years	Female	17	123.2
Case 2	66 years	Male	19.5	97.5
Case 3	53 years	Male	Above 20	Above 200
Case 4	65 years	Male	0.25	155.9

After a comprehensive analysis of various factors and findings in each case, with valid importance given to history, biomechanics of related injuries, other autopsy findings, and point-of-care test reports, a final opinion regarding the cause of death was given (Table 2). 

**Table 2:** Final cause of death attributed to cases based on autopsy findings and POCT findings. POCT: point-of-care testing.

	Cause of death
Case 1	Head injury and air embolism
Case 2	Acute myocardial infarction
Case 3	Acute myocardial infarction
Case 4	Head injury

## Discussion

This case series explores four different forensic cases, each discussing case-specific factors such as history, clinical findings, autopsy findings, traumatic injuries, and cardiac pathology. POCT in forensic settings refers to tests performed during autopsy examination to facilitate forensic surgeons in determining the cause of death. In this case series, the autopsy surgeons utilized the SD Biosensor F2400 Analyzer (Suwon, South Korea), a fluorescent immunoassay-based semi-automatic system, and the POCT cartridges for cTnI and CK-MB. The procedure was conducted by mixing 100 µl of peripheral venous blood with an extraction buffer and by conducting the test with the F2400 Analyzer. The complete procedure, including the CK-MB test and the cTnI test, was taken within 10 minutes and was achieved while an external examination of the deceased was conducted. This ensured that no extra time was dedicated to the POCT examination. Also, in all these cases direct cardiac blood sampling was not preferred to avoid any intra-cardiac events, alternative sampling sites such as the supra‐clavicular and infra‐clavicular approaches for subclavian vein [4], peripheral venous blood samples (from femoral veins) were obtained. 

The specific analytical characteristics of the CK-MB POCT device were significant. As reported in the manufacturer's extended technical documents, the limit of blank (LOB) exhibited by the device was 0.337 ng/mL, which signified the maximum possible value of a blank sample [5]. The LOD, or the limit of detection that could reliably be distinguished from the blank, was measured at 0.555 ng/mL. The limit of quantitation (LOQ), which indicates the lowest concentration for which the measurements are accurate, was measured at one nanogram per milliliter. All of these values are significant. The LOB indicates the minimum or the baseline level, the LOD indicates how sensitive the test is to detect low concentrations, and the LOQ is the lower limit for the exact quantitation of the assay. As for the coefficient of variation (CV) % values of cTnI POCT, as reported by the manufacturer, the quality control low (QCL), quality control medium (QCM), and quality control high (QCH), and the precision of the test at three levels, namely low, medium, and high, were 8.8%, 7.4%, and 7.1%, respectively [5]. Lower values of CV % at higher concentrations indicate higher precision, thereby enhancing the test’s capability to accurately measure those samples with higher levels of biomarkers [5].

Regarding myocardial infarction, CK-MB concentration increases tremendously in four hours to nine hours after the chest pain. It then peaks at 10 hours to 24 hours and returns to normal after two days to three days. As told above CK-MB is a very sensitive and an early marker to detect and monitor the effect/ injury happening in myocardial muscles. Its elevation is highly valuable and an excellent clinical application for myocardial infarction. On the other hand, cTnI increases only after some hours of ischemic injury happening to the myocardial muscles. It peaks in the blood vessels within 12 hours to 48 hours showing the injuries in myocardial muscles and remains in the body for the next several days [6]. The use of two different biomarkers: CK-MB and cTnI not only increases diagnostic accuracy but also decreases the chance of both being false positive, thus reducing false increases or rise of any one biomarker by causes other than cardiac injury.

In Case 1, a road traffic accident, there is a mixture or a combination of so many types of injuries, the upshots of which show antemortem head injury and air embolism as the causes of death. The presence of air embolism within dural vessels and heart chambers adds a layer of complexity that increases the difficulty of the case during dissection and eventually causes difficulties in the cause of death identification. The air embolism leads to cardiac injury thereby increasing the concentration of both cTnI and CK-MB in the blood. The particular kind of air-filled heart chambers has been seen and reported by many authors in cases of head injury. However, the myocardial injury effect has not been mentioned or studied by any specific author [7].

In Case 2, a 66-year-old man expired due to myocardial infarction caused by coronary artery occlusion. The correlation between clinical symptoms, autopsy findings, and diagnostic biomarkers such as cTnI, and CK-MB illustrates the multidimensional nature of cardiovascular cases. In the absence of any history of cardiac disease and with vague nonspecific symptoms prior to death, this case could have resulted in a missed cardiac pathology leading to an obscure or negative autopsy, which is a scenario where a routine autopsy may fail to reveal a definite cause of death. This is considered as such because these types of cases do not reveal any gross or histopathological findings in investigating the case of death other than a partially blocked coronary artery mostly due to the limited time gap between the onset of the terminal event and the death of the individual. It has been discussed by several authors that cardiac markers such as cTnI and cTnT can be employed postmortem, however, to the best of our knowledge, they are all through laboratory analysis. Since it is a time-consuming activity, it cannot generate rapid results by any means, which is contrary to the case of this case series where POCT was administered [8-10].

In Case 3, a revisit to the reported Case 2, there is emphasis on the complications associated with sudden collapse. Comprehensive postmortem findings indicated that the deceased had coronary artery occlusion, old and recent infarcts, and no external injuries. The value of cTnI and CK-MB diagnostic tests in our case was not inconsiderable: the importance of forensic criteria was to determine the manner of death. The collapse that transpired suddenly and without notice on any occasion, coupled with the findings, gave a mixed picture. As the cTnI POCT value was more than 20 and the CK-MB POCT value was more than 200, it implied an acute cardiac affair. This case had very vivid gross findings and the POCT results gave a compelling cause of death and also helped the authors to rely more on POCT modalities for giving accurate results on postmortem samples.

Finally, Case 4 describes a 65-year-old man who died due to a head injury sustained in a fall from stairs. The postmortem examination revealed a variety of injuries including skull fractures and extensive brain trauma. While the increase in CK-MB represents some difficulty in determining whether it was caused by severe trauma or represents a degree of cardiac involvement during the severe trauma death, it still demonstrates the complex interactions between traumatic injuries and heart pathology, which determine complex unnatural deaths. This final case stresses the importance of cardiac biomarkers and expands understanding of the difficulties forensic laboratories may encounter when performing tests related to traumatic injuries emerging at the same time as cardiac events. This case also validated our approach to using two biomarkers instead of one to avoid false positive interpretations of cardiac pathology.

Cardiac troponin I and T are markers for myocardial injury that are sensitive and specific. They are released into the bloodstream due to cardiac muscle injury. It rises early within hours of injury, peaks in 12-48 hours, and persists for several days. Though CK-MB also represents cardiac muscle injury, it rises and falls quickly and is hence useful in the early detection of the severity of myocardial infarction. However, cTnI and cTnT are considered more reliable markers for longer duration as they rise and sustain to detectable levels for longer duration [9,11-13].

High-sensitivity cardiac troponin I (hs-cTnI) and conventional cTnI are used as biomarkers to evaluate cardiac damage. Due to its increased sensitivity, hs-cTnI detects lower levels of myocardial injury; it also has a faster rise and fall than the use of conventional cTnI. In this regard, hs-cTnI is useful as an earlier detection of cardiac events, though the conventional may be relevant today, when precision in the low ranges is unimportant, as in death investigations. Overall, the use of high-sensitivity assays has revolutionized cardiac diagnostics, providing more insight into myocardial injury dynamics [14,15].

When reading the results and drawing conclusions regarding the injury site, it is vital to remember that elevated levels of CK-MB may not always refer to cardiac-specific issues. If a patient suffers a head injury, especially one severe enough to leave an impact, the CK-MB levels can also increase because this enzyme is present in the skeletal muscles as well. When one receives a significant head injury, the trauma can also be associated with chest and torso injuries, which means that the CK-MB is released into the serum because the skeletal muscle is damaged. Therefore, it is crucial to consider the occurrences, that are not cardiac, and that may elevate the levels of this enzyme as a part of a broader clinical examination of the individual and potential sources of muscle damage or injury. This also explains that in the autopsy of Case 4 of this case series, cTnI levels did not rise, because the patient had a head injury and trauma to the skeletal muscles but the CK-MB levels were elevated [12].

This article highlights the importance of POCT in forensic pathology, specifically in quickly capturing cTnI and CK-MB, which are important cardiac biomarkers. This series of cases highlights the accuracy with which POCT detects myocardial injury, even in situations that are complicated by trauma and an overlapping cardiovascular event. The rapid turnaround of POCT enables real-time diagnostic insights during the "golden hour," the critical one- to two-hour window while the autopsy is actively being conducted. This timely information empowers the autopsy surgeon to either identify vital pathological evidence or confidently rule out organ involvement, thereby guiding the examination with precision. In the absence of POCT, such opportunities are lost, as retrospective retrieval or re-examination of organs is no longer possible once the autopsy is complete. This makes POCT not only a tool of convenience but also a decisive aid in preventing missed findings within the constraints of the forensic timeline [9,16]. The unequivocal association between clinical symptoms, autopsy data, and POCT makes these tests an appropriate means for the rapid and certain identification of the cause of death in cases like this one, which could empower POCT as a novel potent technique within modern forensic pathology.

As the cause of death in all four cases was conclusively established through detailed autopsy examination, further toxicological and histopathological investigations were deemed unnecessary, as they would have added an undue burden on the already overburdened forensic science laboratory without contributing additional diagnostic value.

Following death, the biochemical milieu within the body alters dynamically with inevitable ramifications on postmortem concentrations of various biochemistry analytes [17]. These markers can be greatly affected by terminal events, reflecting the immediate pre-death period and agonal end-stage of the dying process [18]. Agonal influences, including hypoxia or circulatory collapse, could affect the levels of biomarkers [19]. In addition, the usage of medical drugs prior to death may contribute to further variability in postmortem biochemical profiles [20]. Accurate interpretation in forensic investigation relies on an understanding of these dynamics. Herein lies the challenge of distinguishing antemortem changes from postmortem alterations, unless close attention is paid to how terminal events and medical interventions can affect biochemistry markers hence emphasizing the need for meticulous analysis of each case.

## Conclusions

Utilizing point-of-care tests for cardiac biomarkers may be considered a valuable addition to the interpretation of postmortem changes that may be used for the further development of forensic pathology. The point is that it was evident from the cases described in this study that processes caused by trauma or some cardiovascular events and the application of separate tests such as cardiac troponin I and creatine kinase-myocardial band may help in evaluating the situation more carefully and distinguishing their signs. The advantages of using such testing in postmortem examination are to make the information about the cause of death more defined and the value of complementing the limitations in time that are observed when evaluating gross or histopathology data made during the terminal event. In this way, it is possible to state that the recent development in diagnostics presents an advantage for those cases where timely information is required to make a decision and the new postmortem diagnostic tool is needed to use in forensic pathology for a more detailed evaluation of the case.
